# Ophthalmic examination as a means to diagnose Subacute Sclerosing Panencephalitis: an optical coherence tomography and ultrawide field imaging evaluation

**DOI:** 10.1186/s40662-016-0066-2

**Published:** 2017-01-19

**Authors:** Koushik Tripathy, Rohan Chawla, Kanhaiya Mittal, Rajni Farmania, Pradeep Venkatesh, Sheffali Gulati

**Affiliations:** 1 Department of Retina and Uvea, Dr. Rajendra Prasad Centre for Ophthalmic Sciences, All India Institute of Medical Sciences (AIIMS), New Delhi, 110029 India; 2 Department of Pediatric Neurology, All India Institute of Medical Sciences (AIIMS), New Delhi, 110029 India

**Keywords:** Epilepsy, Measles, Seizures, SSPE, Ultrawide field imaging, Optos, Optomap

## Abstract

**Background:**

Subacute sclerosing panencephalitis (SSPE) is a potentially fatal complication of measles. The authors report a case of recurrent myoclonic jerks under investigation, whose ophthalmic examination pointed to the diagnosis.

**Case presentation:**

A 12-year-old boy with recurrent episodes of myoclonic jerks was found to have optic disc pallor and an irregular macular scar with pigmentation in the left eye. The retinal finding proved to be a strong diagnostic clue for SSPE. There was a history of exanthematous fever in childhood. Antibodies against measles were detected in both the cerebrospinal fluid and serum. Retinitis with intraretinal and subretinal hemorrhage in the right eye was noted 6-weeks after the initial presentation.

**Conclusion:**

The authors describe the importance of ophthalmic evaluation in cases of recurrent myoclonic jerks. Optical coherence tomographic features and ultrawide field imaging characteristics of a case of SSPE are described.

## Background

An aberrant measles virus causes subacute sclerosing panencephalitis (SSPE). This disorder causes significant neurological morbidity and has a high mortality rate [1]. The eye is involved in up to half of the cases [1]. SSPE continues to be clinically relevant in this era of immunization, especially in the developing countries [1]. We report a case of a 12-year-old boy in whom the fundus picture helped to reveal the primary diagnosis. The patient subsequently developed a hemorrhagic retinitis in the fellow eye.

## Case presentation

A 12-year-old boy presented to the neurology division, department of pediatrics, with episodes of seizures for the past 10 months. Seizures were generalized tonic clonic for the first 3 months, then the child developed recurrent episodes of myoclonic jerks 7–8 episodes per day with frequent falls. Six months after the onset of seizures, he developed disruptive and aggressive behavior along with poor scholastic performance. Subsequently, he developed progressive visual loss in the left eye (LE), six weeks prior to presentation. The provisional diagnosis by the pediatric neurologist was a neurodegenerative disease with myoclonic epilepsy under investigation.

**Fig. 1:**
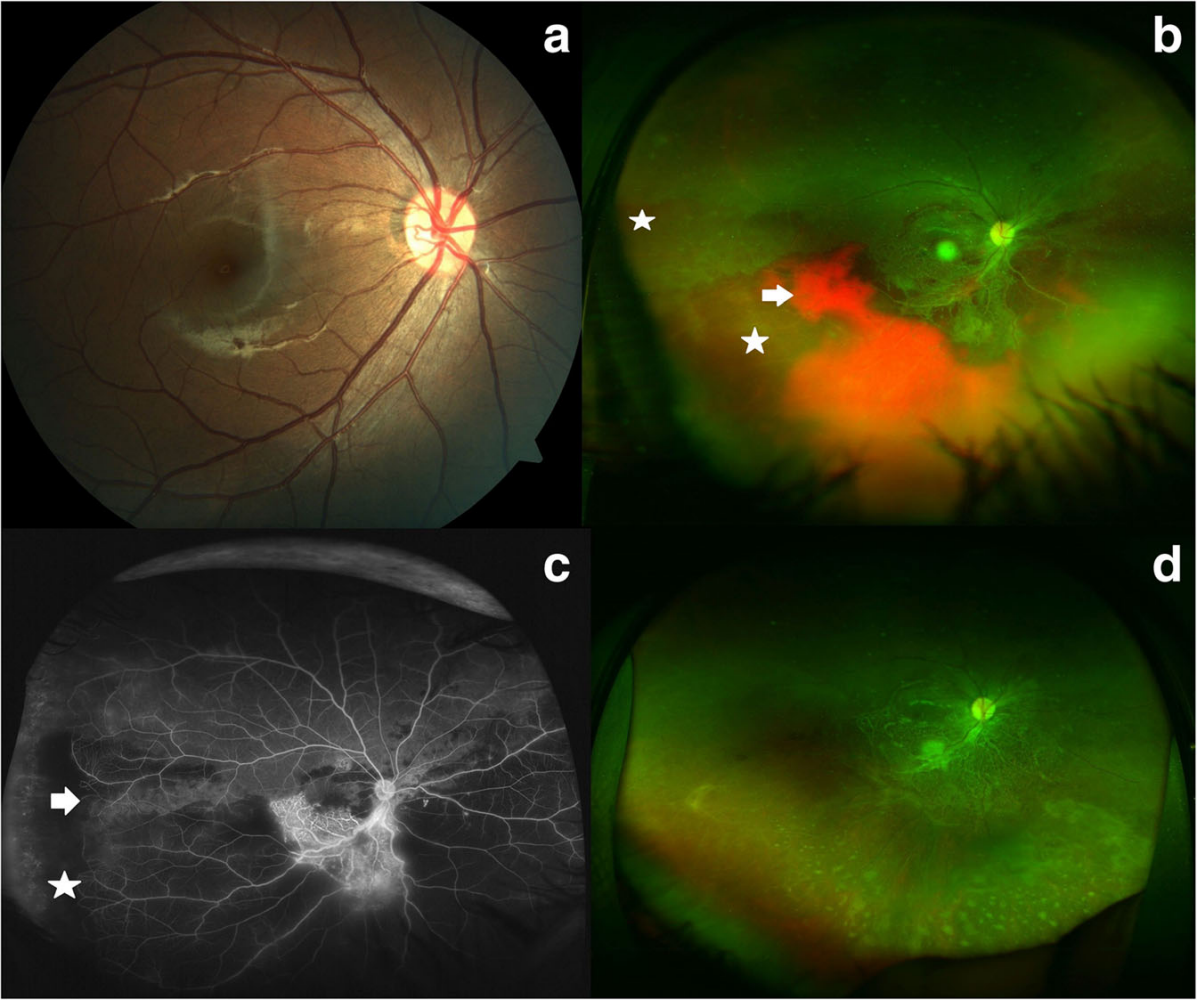
The evolution of clinical findings in the right fundus. **a** The right fundus showed mild temporal pallor of the optic disc. **b** The right eye, at six weeks after initial presentation, showed thinning of the retina at the posterior pole, with inferior intraretinal (*arrow*) and subretinal (*star*) hemorrhage. **c** The ultrawide field fluorescein angiogram (UWFA) of the right eye revealed hyperfluorescence along the inferior arcade and blocked fluorescence due to the hemorrhage. Peripherally there were dropouts of vessels (*arrow*) and arteriovenous loops (star). **d** The hemorrhage resolved leaving behind a moth-eaten retina with conspicuous retinal vessels and inferior peripheral subretinal deposits

**Fig. 2:**
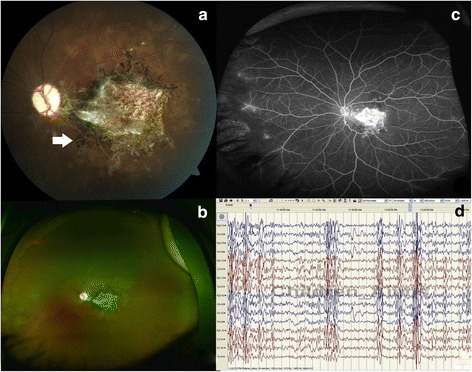
Follow-up of the changes in the left eye and the electroencephalogram. **a** The left eye at the first ophthalmic evaluation showed macular scar with pigment splinters (*arrow*). Small irregular whitish lesions with some pigmentation around the macular scar suggest distinct areas of chorioretinal atrophy. **b** The ultrawide field fundus photograph of the left eye 6 weeks after the initial presentation to the Ophthalmology department showed similar findings as in the presentation. **c** The ultrawide field fluorescein angiogram done at that time showed window defect at the macular, nasal capillary non-perfusion, and paravascular staining. **d** The electroencephalogram showed generalized pseudo-periodic epileptiform discharges at variable intervals of 6–30 s (compressed image at paper speed of 10 mm/s)

On examination, the child had multiple scar marks on the forehead due to recurrent falls. He was oriented with normal speech but had poor memory and insight. The boy was referred to the Ophthalmology department for fundus evaluation. The visual acuity with a pinhole in the right eye (RE) was 6/9 and in the LE was 3/60. A left relative afferent pupillary defect was present. Only mild pallor of the right optic disc was discernible (Fig. 1a). The left optic disc was pale and the macula showed a subretinal scar with radial pigment splinters [2] (Fig. 2a, arrow). Further inquiry revealed that he was unvaccinated and had a history of measles at two years of age in the form of exanthematous fever.

**Fig. 3:**
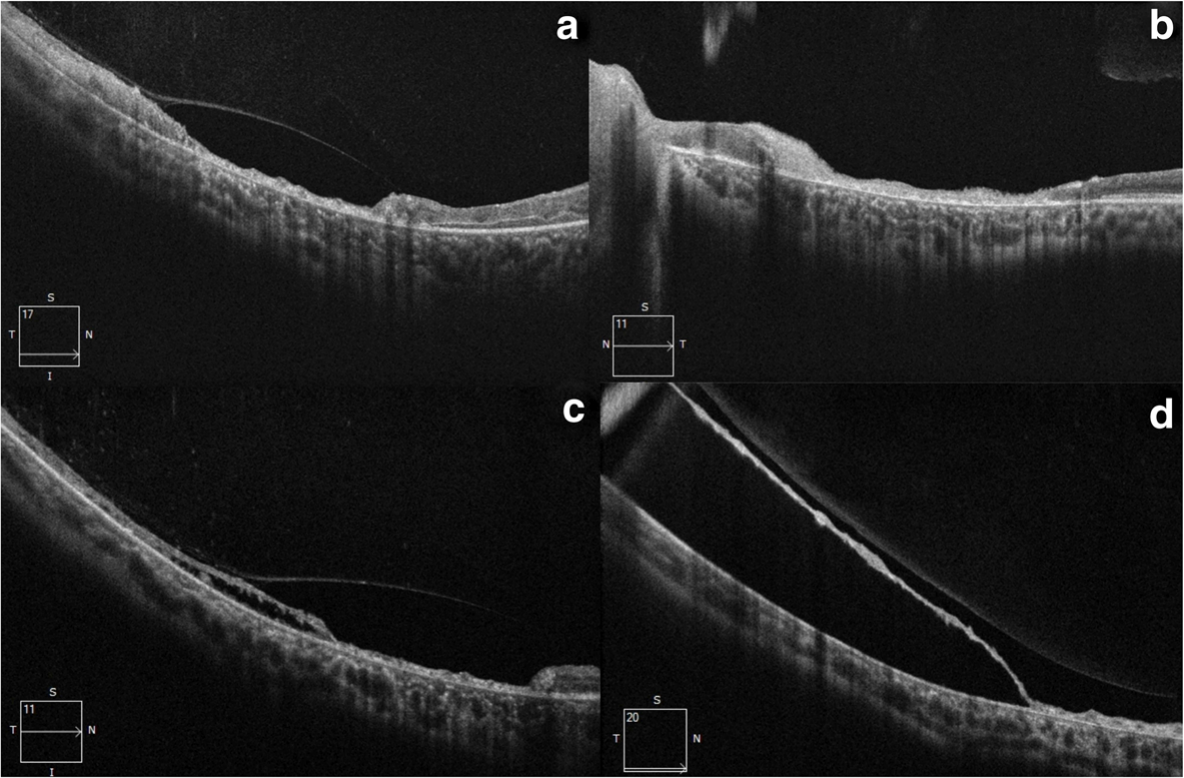
Optical coherence tomography features of the eyes. **a** The optical coherence tomography (OCT) of the right eye at six weeks after initial presentation showed thinning of the retina with a detached internal limiting membrane. **b** The left eye revealed severe retinal thinning at the posterior pole. **c** The OCT of the right eye one week after the appearance of hemorrhage showed necrosis of the outer retina with resulting cavitation. **d** The OCT of the inferior retina of the right eye after the intraretinal and subretinal hemorrhage resolved (3 months) showed a persistence of a continuous thin inner retinal layer with subretinal fluid

On further evaluation, magnetic resonance imaging (MRI) of the brain was found to be normal. Electroencephalogram (EEG) showed generalized pseudo-periodic complexes (Fig. 2d). Measles ELISA-IgG antibody was positive in both the cerebrospinal fluid (CSF) and serum confirming the diagnosis of SSPE. The titer of anti-measles antibody in the serum and CSF was 1:256 and 1:16, respectively [1]. The child was started on isoprinosine and anti-epileptic drugs. Six weeks later, the child presented again with new complaints of reduced vision in his RE. The visual acuity of the RE had decreased to 2/60. The LE maintained status quo (Fig. 2b). Ultrawide field imaging (UWFI) of the RE showed disc pallor, thinning at the macula, sclerosed vessels at the macula and inferotemporal retina along with a hemorrhage in the inferior half of the fundus (Fig. 1b). Part of the hemorrhage was bright red in color (presumably intraretinal, arrow) while part of it was dark red (subretinal, star). The ultrawide fluorescein angiogram (UWFA) of the RE revealed leak from vessels at the posterior pole and paravascular staining, capillary non-perfusion, drop out of retinal vessels (arrow) and arteriovenous loop (star) formation in the temporal periphery (Fig. 1c). The LE showed a window defect at the macula and capillary non-perfusion and staining of few nasal peripheral veins (Fig. 2c). An optical coherence tomography (OCT) inferior to the fovea of the RE showed retinal atrophy with an overlying detached internal limiting membrane (Fig. 3a). The LE revealed retinal thinning of the macula (Fig. 3b). Within one week, the inferotemporal part of the right macula showed cavitary changes in the outer retina suggesting a necrotizing outer retinitis (Fig. 3c). The subretinal hemorrhage completely resolved in the RE by 3 months leaving a moth-eaten thinned out retina, conspicuous retinal vessels, and peripheral subretinal deposits (Fig. 3d). The OCT of the RE showed a continuous thin strip of the inner retina with underlying fluid and absence of outer retinal layers along with macular atrophy (Fig. 1d). The visual acuity in the RE was 1/60 at this point of time. Unfortunately, the boy expired 30 weeks after initial presentation.

## Discussion

Myoclonic seizures may be seen in a myriad of conditions [3, 4] including inherited causes such as progressive myoclonic epilepsies or acquired causes due to anoxia, tumors, metabolic encephalopathies (especially uremia), degenerative central nervous system disease and/or viral infections. Progressive myoclonic epilepsies are inherited genetic epilepsies, which consists of various disorders namely Unverricht Lundborg disease, neuronal ceroid lipofuscinosis (NCL), Lafora body disease (LBD), myoclonic epilepsy with ragged red fibers (MERRF), dentato-rubro-paladal-Lysian atrophy, Sialidosis, and Juvenile GM2 gangliosidosis. The provisional diagnosis by the pediatric neurologist in the current case included relatively common diseases such as SSPE, progressive myoclonic epilepsies likely LBD, MERRF, Juvenile GM2 gangliosidosis, or NCL. Typical ocular findings are pathognomonic of many diseases associated with myoclonus e.g., the presence of cherry-red spot at the fovea would suggest Tay Sach’s disease, sialidosis, and other lipid storage disorders. In addition, pigmentary retinopathy is known to occur in NCL and MERRF. Fundus is usually normal in Lafora body disease.

In our case, presence of a unilateral non-excavated macular scar with pigment-splinters led us to suspect that it was SSPE. Dyken’s diagnostic criteria [1] for SSPE include typical subacute mental deterioration and myoclonus, characteristic electroencephalographic changes, elevated CSF globulin >20% of total CSF protein, increased CSF measles antibody titers, and typical brain biopsy. EEG in SSPE characteristically shows long duration generalized periodic discharges of at least 4 s. Classically, the interval between complexes is fixed but variable interval between periodic discharges may also be seen, referred to as pseudo - periodic or quasi-periodic discharges [5, 6]. Anti-measles antibody titers of 1:4 or greater in CSF and 1:256 or greater in serum is diagnostic of SSPE [1]. Fundus changes in SSPE are predominantly seen at the posterior pole. Common features include focal necrotizing macular retinitis, pigmentary changes at the macula often simulating heredomacular degeneration, and macular scar formation with internal limiting membrane contracture or dragging of the surrounding retinal vessels [1, 2]. The reported optic disc changes include disc edema (papillitis, papilledema), total disc pallor, temporal disc pallor, and disc gliosis [2, 7]. Other less common reported features include preretinal opacity, periarteriolar sheathing, intraretinal lipid deposition, acute multifocal placoid pigment epitheliopathy-like lesions, macular edema, macular hemorrhage, multifocal subretinal lesions, supranuclear gaze palsy, homonymous visual field defects, cortical blindness, and Balint syndrome [1, 2, 7]. Intraretinal hemorrhage [2, 8–11] and serous macular detachment [12, 13] are also known to occur in SSPE retinitis. It was difficult to explain the pinhole vision of 6/9 in the RE at presentation, from the slit lamp and fundus examination. Though refraction was advised, the patient did not comply for the same. There was only mild optic disc pallor in the RE. A component of mild damage to the optic nerve in this eye at this point in time cannot be ruled out.

Both intraretinal and subretinal hemorrhage in the area of retinitis has not been described before in SSPE to the best of our knowledge. UWFI picked up retinitis extending to the peripheral retina and other peripheral vascular changes in this case of SSPE. The UWFA in our case did not reveal any obvious leak suggestive of retinal neovascularization or choroidal neovascularization. We hypothesize that the hemorrhage may have occurred due to the rupture of the vessels of the deep intraretinal plexus due to the retinitis/necrosis of the retinal vessel walls due to associated vasculitis in the area of the necrotizing retinitis. The vessels of the intraretinal plexus can also rupture due to stretching as can occasionally be seen in intraretinal cysts of old retinal detachments [14]. However, the presence of neovascularization hidden within the hemorrhage cannot be excluded. The peripheral capillary non-perfusion secondary to vasculitis may have predisposed to a retinal neovascularization.

The authors have shown that UWFI can be an important modality to objectively document and monitor cases of peripheral retinitis such as acute retinal necrosis [15]. In this case, UWFA further helped us document the peripheral vascular changes in both eyes. The use of OCT also revealed a loss of outer retinal tissue before thinning of the entire retina, suggesting initial outer retinitis.

## Conclusions

Ultrawide field imaging of more cases in various stages of this disease may further enhance our understanding of the ocular manifestations seen in SSPE. The authors also want to emphasize the role of an ophthalmological examination in cases of seizures of undiagnosed etiology. The typical macular scar with pigment splinters seen in the left eye of our case was highly suggestive of SSPE.

## Abbreviations

CSF: Cerebrospinal fluid
EEG: Electroencephalogram
ELISA-IgG: Enzyme-linked immunosorbent assay-Immunoglobulin G
LBD: Lafora body disease
LE: Left eye
MERRF: Myoclonic epilepsy with ragged red fibers
MRI: Magnetic resonance imaging
NCL: Neuronal ceroid lipofuscinosis
OCT: Optical coherence tomography
RE: Right eye
SSPE: Subacute sclerosing panencephalitis
UWFA: Ultrawide field fluorescein angiogram
UWFI: Ultrawide field imaging
